# Probing the Strength of Infants' Preference for Helpers over Hinderers: Two Replication Attempts of Hamlin and Wynn (2011)

**DOI:** 10.1371/journal.pone.0140570

**Published:** 2015-11-13

**Authors:** Eliala Salvadori, Tatiana Blazsekova, Agnes Volein, Zsuzsanna Karap, Denis Tatone, Olivier Mascaro, Gergely Csibra

**Affiliations:** Cognitive Development Center, Central European University, Budapest, Hungary; University of Bologna, ITALY

## Abstract

Several studies indicate that infants prefer individuals who act prosocially over those who act antisocially toward unrelated third parties. In the present study, we focused on a paradigm published by Kiley Hamlin and Karen Wynn in 2011. In this study, infants were habituated to a live puppet show in which a protagonist tried to open a box to retrieve a toy placed inside. The protagonist was either helped by a second puppet (the “Helper”), or hindered by a third puppet (the “Hinderer”). At test, infants were presented with the Helper and the Hinderer, and encouraged to reach for one of them. In the original study, 75% of 9-month-olds selected the Helper, arguably demonstrating a preference for prosocial over antisocial individuals. We conducted two studies with the aim of replicating this result. Each attempt was performed by a different group of experimenters. Study 1 followed the methods of the published study as faithfully as possible. Study 2 introduced slight modifications to the stimuli and the procedure following the guidelines generously provided by Kiley Hamlin and her collaborators. Yet, in our replication attempts, 9-month-olds’ preference for helpers over hinderers did not differ significantly from chance (62.5% and 50%, respectively, in Studies 1 and 2). Two types of factors could explain why our results differed from those of Hamlin and Wynn: minor methodological dissimilarities (in procedure, materials, or the population tested), or the effect size being smaller than originally assumed. We conclude that fine methodological details that are crucial to infants’ success in this task need to be identified to ensure the replicability of the original result.

## Introduction

The study of sociomoral sensitivity in early infancy is one of the most productive and successful lines of inquiry in the last decade of developmental science. Ever since the seminal studies by Premack and Premack [1] and by Kuhlmeier, Wynn, and Bloom [2], findings about infants’ ability to evaluate the sociomoral valence of actions and agents have been steadily accumulating. A great deal of the empirical evidence supporting the existence of an early developing “moral sense” [3] comes from studies using the so-called “manual choice” paradigm [4]. In the standard version of this paradigm, infants are first exposed to a “morality play” [5] in which a character (the Protagonist) tries to achieve a goal, and, on alternating trials, is either helped by a second character (the Helper) or hindered by a third character (the Hinderer). Once familiarized or habituated to these events, infants are presented with the Helper and the Hinderer and encouraged to choose between them. Typically, it is expected that infants prefer to reach for the Helper over the Hinderer. Part of the appeal and success of the manual choice paradigm rests in its straightforward logic: if infants are capable of differentiating between the two actions in terms of their social valence (on the basis of how they contributed to the realization of the Protagonist’s goal), and differently evaluate each character on the basis of these actions, they should develop character-specific preferences, which would be revealed at test in their reaching behavior. Crucially, for infants to form a preference for the Helper over the Hinderer, they need to attribute to these agents certain properties that go beyond local assessments restricted to the specific interaction observed [6].

The manual choice paradigm proved to be a valuable methodology for charting out the complexities of infants’ socio-moral evaluations. The picture that eight years of research employing this paradigm revealed is that of a remarkably precocious and sophisticated sociomoral sensitivity in infancy. As soon as they begin to reach for objects (by around 5 months of age), infants already show a systematic preference for helpers over hinderers in a variety of contexts. Whether the Protagonist attempts to climb a steep hill, persistently tries to open a box to retrieve a toy, or needs assistance to have her dropped ball returned, infants systematically prefer the agent facilitating the achievement of this goal [4,7,8]. Furthermore, infants’ evaluations are specifically social: infants do not prefer characters that engage in prosocial acts with a non-social agent (e.g., a pair of pliers: [7]). Similarly, infants’ preference for the Helper disappears when the Protagonist’s goal is made ambiguous (e.g., because the Protagonist is gazing to a location different from the one she is attempting to reach: [9,10]). These effects have proven remarkably strong. Summarizing data collected in studies using the hill, ball, and box scenarios, Hamlin [11] reported that 179/215 infants (i.e., more than 83%) chose the helper.

Adding to the remarkable context-dependence of infants’ evaluations, further work demonstrated that infants’ evaluative inferences are not inflexibly linked to either the local valence of the observed action, or the actual production of outcomes compatible with the Protagonist’s goal. For instance, presented with a scenario in which two individuals respectively facilitate and obstruct the goal of another agent who has in turn hindered a third party, infants as young as 4.5 months of age already show a marked preference for the “Punisher” (i.e., the individual who hindered the Hinderer: [12, 13]). Likewise, by 8 months of age, infants prefer characters who try but fail to help over those who try but fail to hinder [14]. Finally, by 10 months, infants prefer Helpers to Hinderers only if both characters knew that their actions were helpful or unhelpful to the acquisition of the Protagonist’s preferred object [15].

Importantly, the manual-choice paradigm has been successfully adopted in domains other than instrumental helping, such as distributive fairness [16], sympathy towards victims [17], and homophily-based favoritism [18], imposing itself as prime method for assessing the full scope and diversity of infants’ early sociomoral competencies.

Given the impact and success of the manual choice paradigm in demonstrating infants’ sociomoral evaluation of agents, we sought to replicate one of the first “morality plays” in which this methodology has been tested. We selected the “box scenario” from a paper by Hamlin and Wynn [7], in which the Protagonist attempts to open a box in order to retrieve a toy, and is either helped or hindered by two characters. Infants were found capable of producing social evaluations when presented with this scenario with varying levels of exposure (from full habituation to single-event presentation), and, remarkably, even when this scenario was nested within a larger “morality play” that required second-order evaluations [13].

Here we report two attempted replications, conducted by two different groups at the same research facility. The first replication followed as faithfully as possible the methodological details reported in Hamlin and Wynn [7]. The second attempt introduced slight modifications to the stimuli and the procedure following recent guidelines generously provided by Hamlin and her collaborators.

## Study 1

Infants were habituated to a live puppet show, during which a character (the Protagonist) was helplessly trying to open the lid of a transparent plastic box to retrieve a rattle placed inside. The Protagonist was alternately either aided or thwarted by two characters, who helped to open the box (the “Helper”) or slammed the box lid closed (the “Hinderer”), respectively. Once the child habituated to the prosocial and antisocial events, habituation trials were terminated and followed by a test, which consisted in presenting infants with the Helper and the Hinderer, and encouraging them to choose one of them by reaching for it.

### Methods

The methods of this study followed the description provided by Hamlin & Wynn [7] as closely as possible. This research (Studies 1 and 2) was approved by the Hungarian Ethical Review Committee for Research in Psychology (EPKEB; code: 2014/6). Participants were recruited through mailings, and were given a toy as gift for participating. In both Studies, informed written consent was obtained from parents of participating infants before the study. Parents were free to withdraw their consent at any time, and the data collected were kept confidential.

#### Participants

Twenty-four full-term, normally developing 9-month-old infants took part in the study (mean age: 8 months 29 days; range: 8;16–9;22). The sample-size was set to ensure stronger statistical power than that of the original study (in which 16 participants were tested). Twenty-three additional infants were excluded from analyses, due to: fussiness (*n* = 3); parental interference (*n* = 1), failing to choose either puppet (*n* = 7), choosing both puppets (*n* = 3), procedural error (*n* = 8), or equipment failure (n = 1). (See S1 Appendix for further details about excluded infants.)

#### Apparatus and Setting

Stimuli were presented to infants on a puppet-theatre stage (60 x 80 cm). A dark blue curtain, which could be raised and lowered by means of a pulley, covered the opening of the stage before and in-between events. A transparent plastic box (15 x 20 x 7 cm), a rattle, and three puppets were involved in the events. A lion puppet played the role of the Protagonist, and two dog puppets, one wearing a red shirt and the other wearing a blue shirt, played the Helper and the Hinderer roles (varied across infants).

Three experimenters were present in the room. E1 was in charge of the online coding of infants’ looking behavior; E2 was in charge of performing the puppet show; and E3 was in charge of instructing the parents, performing the familiarization and the manual choice test, and operating the stage curtain during the habituation phase. E1 was visible to the infants only while entering the room, being otherwise separated from the testing area by means of a wide curtain. E2 was never visible to the infants, as she was fully hidden behind the stage. She was also wearing black gloves to prevent infants from being distracted by her hands appearing from behind the curtains during the puppet manipulation. E3, who interacted with the infants before the study, was hidden behind the stage during the habituation phase, and only reappeared for the manual choice phase. Crucially, only E2 was aware of the identity of Helper and Hinderer, since E3 had no visual access to the puppets during the habituation phase, and E1 monitored the habituation events via a monochrome camera that did not allow discriminating the two puppets on the basis of their t-shirt color. Three cameras, hidden to the infant’s sight, one pointing to the stage, and two on the infant (lateral and frontal view), were used to record the participants during the habituation and the manual choice phases.

#### Procedure

The study consisted of three phases: familiarization, habituation, and manual choice. Before entering the testing room, parents were instructed to keep their eyes closed throughout the habituation and manual choice phases and to avoid interacting with the infants, unless explicitly told so by E3. We let parents soothe their infants briefly during the inter-trial pause in the habituation phase (in which the curtain stage was down for 5–10 s) if the infants started showing clear signs of distress. Once in the testing room, parents were instructed to sit the infants on their laps, 100 cm from the stage.

***Familiarization***: Infants were presented with a transparent plastic box containing a rattle. E3 approached the infant, holding the box in front of their faces, and said, “Look!” (shaking box), “Look!” (grabbing the edge of the box), “Look!” (opening the box), and “Ooh!” (lifting the toy out of the box and shaking it). Afterwards, she said, “Should we put it in again? Look!” (putting the toy back in the box), “Look!” (closing the lid), “It’s in there again!” (shaking the box). Finally, she said, “Should we take it out again?” and repeated the familiarization once more.

**Fig 1:**
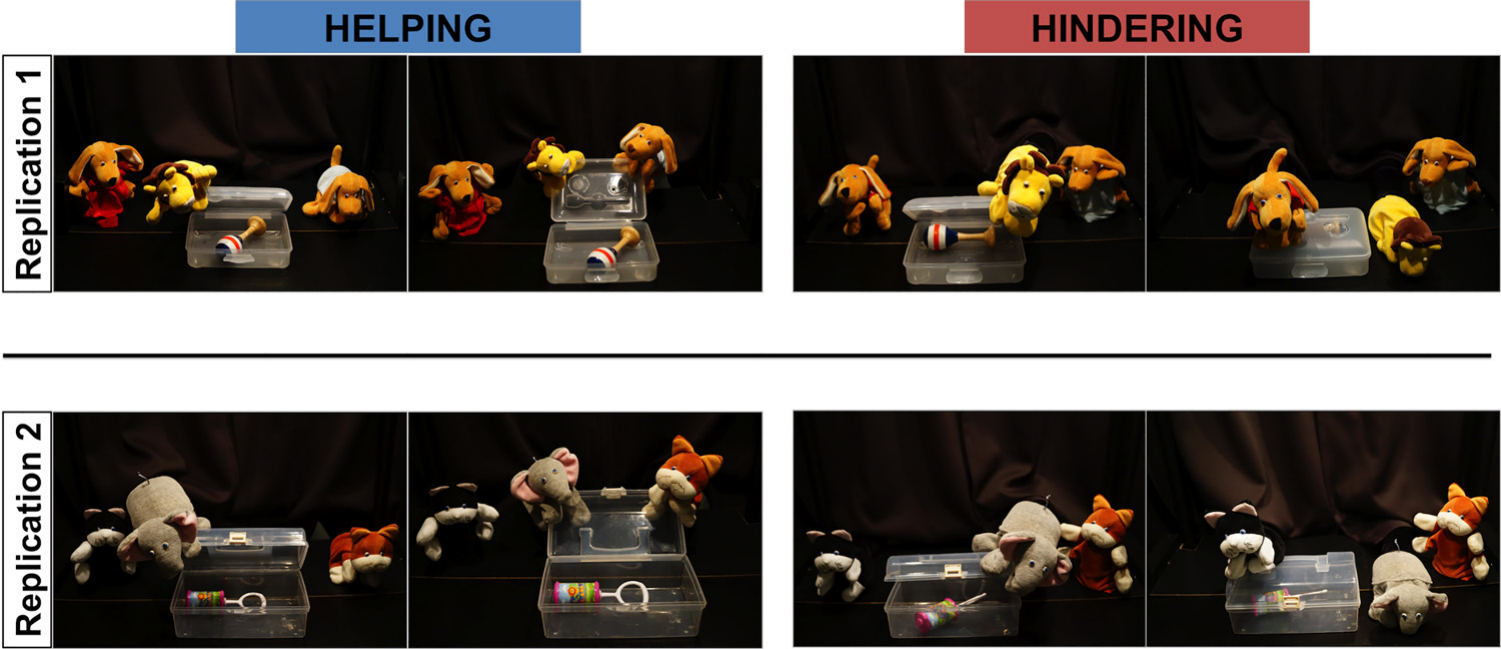
Helping and hindering trials in Studies 1 and 2. Pictures on the left of each panel show the protagonist trying to open the box. Pictures on the right of each panel show a helping or a hindering event.

***Habituation phase***: Each trial of the habituation phase started with raising the curtain, revealing the stage with the rattle in the transparent box in the middle and the two dogs sitting at the back corners of the stage, equidistantly from the box. The Protagonist entered from the center of the back of the stage, in between the two dogs, moving to one side of the box, and looking twice at the toy inside. The Protagonist then jumped on the front part of the lid and tried to open the box five times. In the first two attempts, the Protagonist shook the lid before dropping it shut. In the third and fourth attempts the Protagonist lifted and lowered the lid down while continuously holding it. In the fifth attempt, one of the two dogs either helped or hindered the Protagonist’s attempt to open the box (see Fig 1). During *Helping trials*, the Helper dog grabbed a corner of the lid, and the two puppets opened the box together. Afterwards, the Protagonist reached for the toy inside the box, and stopped there, while the Helper ran off stage. During *Hindering trials*, the Hinderer dog slammed the box lid closed, preventing the Protagonist from getting the toy from the box. The Protagonist laid down next to the box without the toy, while the Hinderer ran off stage. Infants were shown Helping and Hindering events alternately. The average duration of each event was approximately 15 s.

At the end of each event (i.e., as soon as the Helper/Hinderer ran off stage), infants’ looking to the still stage was measured online by E1 using Psyscope. The trial ended when infants (a) looked away for 2 consecutive seconds, or (b) 60 s elapsed. At this point the curtain was lowered by E3 in response to a vocal signal whispered by E1. The inter-trial interval lasted until E2 prepared the stage for the following trial (5 to10 s). Habituation was reached when (1) the sum of infants’ looking time at three consecutive trials was less than half of the sum of their looking time at the first three trials, or (2) a maximum of 14 trials was completed. To indicate to E3 that the infant reached habituation, E1 whispered a second vocal command.

***Manual choice***: E3, who was blind to the identity of the puppets, came out of the curtains, hiding the two dog puppets behind her back, and asked parents to rotate their chairs about 30 degrees to their right. E3 kneeled down at about a meter away from the infant, and said ‘Hi’ in order to get the infant’s attention. Then she showed to the infants the two dogs simultaneously while saying ‘Look!’ Puppets were held at about 50 cm away from infants, and kept still until a choice was made.

In order for a choice to be considered valid, infants had to (a) look first at both puppets; (b) perform a visually guided reach; and (c) show a preference for only one puppet within 40 s. In case infants failed to look at both puppets, E3 slightly wiggled the puppets and repeated the hiding-revealing procedure one more time. Since no information about the time limit for producing a manual choice was provided by Hamlin & Wynn [7], we arbitrarily selected a 40-s limit. This upper limit was sufficient for most infants to produce a choice. Excluding those who did not try to reach for either puppet (*n* = 7), the remaining infants took on average 9.58 s to express their choice (SD = 7.13). The choice was independently coded offline by E1, E2, and a third coder blind to the identity of the puppets. The inter-coder agreement was 100%.

Across infants, the following three factors were orthogonally counterbalanced: (1) identity of the puppets (Helper wearing red or blue shirt), (2) order of events during habituations (Helper first or second), and (3) side during habituation (Helper left or right). In addition, (4) the puppets' side during manual choice was equalized (Helper on the left or right side for half of the infants).

## Results and Discussion

All the statistics reported in this paper are two-tailed. The infants habituated in an average of 8.75 trials (*SD* = 2.29 trials). During the manual choice test, infants did not significantly prefer the Helper over the Hinderer puppet (15 infants out of 24, *p* = .307, binomial test). Infants’ choice was not significantly influenced by the puppets’ t-shirt color (13 infants out of 24 selected the puppet wearing a blue shirt, *p* = .838, binomial test), or by the puppets’ location at test (15 infants out of 24 selected the puppet located on their left side, *p* = .307, binomial test).

Infants showed no significant preference for the Helper over the Hinderer in Study 1. After this first attempt, we contacted Kiley Hamlin, who generously provided detailed and helpful advice that we used to set the procedure for Study 2.

## Study 2

To keep the two replication attempts relatively independent from each others, new materials were used and a new team of three experimenters ran Study 2. E2 and E3 of this study were informed about the results of the first replication attempt only after completing the experiment.

### Methods

The method of this study was almost the same as of Study 1, but we refined the procedure using the instructions we received from Kiley Hamlin (personal communication).

#### Participants

Twenty-four 9-month-old full-term, normally developing infants participated (mean age: 9 months, 0 days; range 8;18–9;25). Fifteen additional infants were excluded from analyses because of fussiness (*n* = 6), equipment failure (n = 1), procedural errors (*n* = 3), making no identifiable choice (*n* = 3, see Procedure), parental interference (*n* = 1), or because the participant fell asleep (*n* = 1). (See S1 Appendix for further details about excluded infants.)

#### Apparatus and Setting

A new transparent box (size 12 x 22 x 10 cm), a novel rattle, and a different set of puppets were used. An elephant hand puppet played the role of the Protagonist. Two cat hand puppets that differed only in color (black vs. orange) were used as Helper and Hinderer. Following advice from K. Hamlin and her team, the stage curtain was decorated with a few small animal stickers to make it more infant-friendly. Moreover, an additional video camera was installed to get a better view of the infants’ behavior during manual choice.

#### Procedure

We introduced several procedural modifications based on advice from Kiley Hamlin and her team. First, background music was played softly throughout the experiment. Second, a “curtain familiarization” was added to make infants comfortable with the operation of the stage curtain. Third, the manual choice procedure was revised using a detailed script provided by Kiley Hamlin (personal communication).

***Familiarization***: After the box familiarization (ran as in Study 1), E3 familiarized infants with the curtain’s movements by raising it and lowering it two times. While raising the curtain, E3 shook a toy containing a small bell and said: “The curtain is going up.” While lowering the curtain she said: “The curtain is going down.” At the end of familiarization, E3 disappeared behind the stage and the habituation procedure started.

***Habituation phase***: The procedure matched to that of Study 1, except that E1 signaled the end of each habituation trial and of the entire habituation phase with a visual signal instead of whispering vocal commands.

***Manual choice***: The procedure for this phase was thoroughly revised based on detailed instructions and video examples provided by Kiley Hamlin. The test started by E3 coming out from behind the curtain, holding the puppets behind her back. She asked the caregiver to turn their chair to face her (a rotation of about 30 degrees to the caregiver’s left). Moreover, E3 checked that the infant was appropriately positioned, and if necessary she asked the caregiver to make corrections. The caregiver had to place the infant close to her knees and to hold her at the level of her chest so that the infant would sit straight and would be able to move her arms without leaning on his/her parent’s body. Instructions about how to position the infant during test were given before the experiment to reduce the delay between the end of habituation and the manual choice test. Once the infant was appropriately positioned, E3 kneeled down at about 1 meter in front of him/her. She said “Hi [name of the baby]”, and established eye contact with the infant. Once this was achieved, E3 pulled the puppets from behind her back and said, “Look!” She held the puppets by their back so that her hands were not visible to the infant during choice. E3 placed both puppets at equidistance from the infants, at their eye level and out of their reach. At this point, E3 waited for the infant to glance at each puppet. If after about 3 seconds the participant looked only at one puppet or at her face, E3 gently shook both puppets simultaneously to attract infant’s attention to them. Once the infant had glanced at each puppet, E3 stopped shaking the puppets. E3 then attracted the infant’s attention to herself by moving her head slightly forward, saying “Hi,” and pulling the puppets about 10 cm back. E3 re-established eye contact with the participant, and said: “Who do you like?” She then positioned the puppets for the choice, moving them within the infant’s reach by extending her arms. While moving the puppets towards the child, E3 stopped making eye contact and moved her gaze towards the infant’s chest. Puppets were held about 50 cm apart from each others.

For a contact between the infant and a puppet to count as a choice, the infant had first to look at one puppet, and then reach for this puppet while still looking at it. Thus, touching one or both puppets without looking at them did not count as a choice. Conversely, if infants looked at one puppet and then reached for both while continuing to look at the same puppet, this counted as a choice. If infants did not make a choice within 60 seconds, E3 pulled back the puppets. She then repeated the choice procedure from the moment she pulled the puppets from behind her back. Infants who produced no choice after 60 additional seconds during this second attempt were coded as making no choice. The choice was independently coded offline by E1, E2, and by a third coder blind to the identity of the puppets. The inter-coder agreement was 100%.

The variables counterbalanced in Study 2 were the same as in Study 1.

### Results and Discussion

Infants habituated in an average of 9.00 trials (*SD* = 2.69). During the manual choice test, infants did not significantly prefer the Helper over the Hinderer (12 infants out of 24: *p* = 1.161, binomial test). Similarly, infants displayed no tendency to prefer the black over the orange puppet (14 infants out of 24, *p* = .541, binomial test). Infants showed a marginally significant tendency to select the puppet located on their right side during test (17 out of 24 infants, *p* = .064, binomial test).

## General Discussion

We made two attempts to replicate the results of Hamlin and Wynn [7], without success. In the original study, 75% of infants preferred helpers over hinderers. In our replication attempts, this preference was lower (62.5% and 50%, respectively in Studies 1 and 2), and did not reach statistical significance. Two types of factors could explain why our results differed from the ones of Hamlin and Wynn: methodological dissimilarities (in procedure, materials, or the population tested), or the effect size being smaller than originally assumed.

Study 1 followed the methods described in Hamlin and Wynn [7] as scrupulously as possible. Moreover, Kiley Hamlin and her team provided us with extremely helpful and detailed advice that we adopted to fine-tune the procedure for Study 2. We did modify deliberately the procedure of Hamlin and colleagues in only one respect: We asked caregivers to keep their eyes closed not just at test, but also during habituation. We introduced this change as a default procedural routine, and we remain agnostic as to whether it could have had any effect on infants’ performance. While we cannot a priori exclude that in the original study caregivers might have (inadvertently) influenced their infants by attending the habituation events, to our knowledge, there is no evidence for such a subtle and powerful form of social influence in infancy. Since our replication attempts were conducted in a different country and in a different laboratory, this inevitably resulted in further minor changes from the original study, such as population tested (American vs. Hungarian infants); room arrangement (i.e., infants were not seated to a table directly connected to the puppet stage); size of the stage, puppets, and boxes; distance of the participants from the stage (165 vs. 100 cm); and type of puppets.

We also consulted Kiley Hamlin (personal communication) for an expert opinion on what distinguished the original paradigm from our replications. She noted that in her hindering events the protagonist jumped off the box about one second after the slamming action, paused on the ground, and later put its head down (S2 Movie, 00:37–00:40). Described with an intentional gloss, this sequence of events might have suggested that the Protagonist gave up its goal and expressed unhappiness. By contrast, in Study 1, the protagonist fell off the box simultaneously to the slamming action and landed head down on the ground. In Study 2, the protagonist jumped from the box right after the slamming action, and quickly put its head down (S1 Movie, 00:36–00:38). These shorter delays between events might have hindered event segmentation, or might have changed the interpretation of the event, for example, by suggesting that the protagonist was projected to the ground by the slamming action.

Concerning the helping events, Hamlin (personal communication) noted that the impression that the Helper is doing all the work in pulling up the lid (while the Protagonist remains passively holding onto it) is weaker in our studies than in the original one. This may be due to the fact that the lid of the boxes was smaller in our replications than in the original study, resulting in a shorter trajectory of the Protagonist when the box was successfully opened.

Despite our best efforts, we cannot discount the possibility that infants’ performance might have been affected by any of the factors highlighted above, or that our replications may have omitted further crucial methodological details. Importantly, however, none of the above-reported differences between our replication attempts and the original study have been recognized as necessary to infants’ success in the paradigm used by Hamlin and Wynn [7]. If this were the case, the factors responsible for eliciting early social evaluation using the present paradigm would need to be identified and explicitly reported in the future to ensure the replicability of the original results. Infants’ reaching has been used successfully to investigate early socio-moral reasoning a variety of experimental settings (e.g., [4, 16, 17, 19]). However, as noted by an anonymous reviewer, most of the published studies showing evidence for infants’ disposition to reach preferentially for helpers over hinderers have been collected by a single set of researchers [4, 7, 9, 12, 13, 14, 15, 18]. Studies testing the same capacity in other laboratories have yielded mixed results: one study reports positive evidence [20], another study reports null results [21]. This pattern raises the possibility of a ‘lab effect’, i.e. a tendency for one team of researchers to find consistently stronger effects when using a given experimental paradigm (see for instance [22–23]). Lab effects may arise from crucial methodological details being communicated informally within labs, and perhaps unwittingly omitted from the published materials, and thus could be easily overlooked in attempted replication efforts.

Alternatively, we may have found non-significant results not because of minute methodological factors, but because the size of the effect driving infants’ preferences is smaller than what we anticipated. In the study of Hamlin and Wynn [7], 12 out of 16 infants responded in the expected way. Based on this value, we could estimate the power of our replication studies, i.e., the probability to detect the effect of a specified size when it is present. According to this analysis, each of our experiments (N = 24) had a power of 0.77 to find the one-tailed effect that Hamlin and Wynn [7] reported. Collapsed together the samples of the two studies (N = 48), our replication attempts reach a power of 0.96 for obtaining the same effect. In other words, replicating the study with a sample of 48 infants would yield significant results in 96/100 attempts given the expected effect size. Crucially, however, such an analysis assumes that the results of the original study, which was performed by 16 infants, would be sufficient to accurately estimate the effect size of infants’ preference in the entire population. Such an assumption is rarely warranted. The true size of the effect underpinning infants’ preference might be smaller than what could be inferred from the original study, which could explain why we did not find significant results.

## Supporting Information

S1 Appendix Detailed analysis of the excluded infants in Studies 1 and 2. (DOCX)

S1 Movie Habituation events as seen from the infant’s perspective in Study 2. (MOV)

S2 Movie Habituation events used in Hamlin & Wynn (2011). (MOV)
