# Supplementary material for: Probing the Strength of Infants' Preference for Helpers over Hinderers: Two Replication Attempts of Hamlin and Wynn (2011)
Source: PLoS One. 2015 Nov 13;10(11):e0140570. doi: 10.1371/journal.pone.0140570 (PMC4643979; doi:10.1371/journal.pone.0140570)
Supplement: S1 Appendix — (DOCX) [file pone.0140570.s001.docx]

**Appendix**

In the original study of Hamlin and Wynn, three out of 35 9-month-olds were excluded (9%). By comparison our rejection rate was noticeably higher (Study 1: 49%; Study 2: 38%). For this reason, we decided to include a detailed description of our exclusion criteria, and a re-analysis of the results when all participants making a choice are included.

**Study 1**

**Exclusion criteria**

In Study 1, 23 infants were excluded from the analyses for the following reasons:

*Fussiness (n = 3).* These infants cried during the study, which forced us to terminate the procedure prematurely (either before the infant habituated, or before the infant made a choice during test).

*Failing to choose either puppet (n = 7)*. These infants did not perform any choice before 40 s elapsed.

*Choosing both puppets (n =* 3*).* These infants reached for both puppets at the same time. Only one of the three infants performed a choice that could have considered valid according the criteria adopted in Study 2 (i.e., she looked at one puppet and then reached for both while continuing to look at the same puppet). The choice of this single infant was included in the additional analysis (below). Of the two remaining infants, one reached for both puppets while continuously looking at E3, while the other repeatedly shifted her gaze between the two puppets.

*Procedural errors (n = 8).* Cases 1-2: E3 was not blind to the identity of the puppets; Case 3-4: the box detached from the stage, causing the actions of the Protagonist over the box to be performed incorrectly; Case 5: a new experimenter, who received only a quick briefing about the procedure, was used to replace E2 for one infant, but performed the manual choice incorrectly (by positioning herself wrongly with respect to the infant); Case 6: E1 forgot to turn off a monitor (used in a previous study) placed sideways to the infant, which resulted in the infant repeatedly turning toward the brightly lighted monitor during the habituation; Case 7: E2 made the Hinderer close the box in an incorrectly gentle manner to avoid producing any slamming sound that could have potentially startled the infant; Case 8: The infant was tested on a combination of counterbalanced factors for which enough babies had been tested already.

*Parental interference (n = 1).* The mother pulled back the infant while he was attempting to reach for a puppet. After pushing herself away from the mother’s chest, the infant then reached for the other puppet.

*Equipment failure (n = 1).* The video recorder ceased to function before test.

**Analyses including excluded infants**

Out of the excluded participants in Study 1, 9 infants produced an identifiable choice: the participants excluded because of procedural error (8; 3 chose the helper, 5 chose the hinderer), and parental interference (1; chose the helper). When we included these participants in our data analysis, infants did not significantly prefer the Helper over the Hinderer (19 infants out of 33: *p* = .487, binomial test).

**Study 2**

**Exclusion criteria**

In Study 2, 15 infants were excluded from analyses for the following reasons:

*Fussiness (n = 6).* These infants cried during the study, which forced us to terminate the procedure prematurely (either before the infant habituated, or before the infant made a choice during test).

*Equipment failure (n = 1).* The video recorder ceased to function before test. Although we could record the infant’s choice during the test, we had no way to double-code it.

*Procedural errors (n = 3).* Case 1: E2 inverted the roles of the puppets in one habituation trial (the Helper became the Hinderer); Case 2: The parent was very tall, thus the infant ended up very close to the puppet when placed at the tip of her knees during the choice procedure. As a result, the infant grasped both puppets at once. Case 3: The infant was tested on a combination of counterbalanced factors for which enough babies had been tested already.

*Making no identifiable choice (n = 3)*. Case 1: The infant repeatedly reached for both puppets, without looking clearly at any of them before reaching. Case 2: The infant reached for one puppet without looking at it, and held it for about 15 seconds. When given a second chance to make a choice, the infant reached again for the same puppet without looking at it. Case 3: The infant lost balance, and clung to one puppet to avoid falling.

*Parental interference (n = 1).* The mother opened her eyes and talked to her infant during the manual choice test. Although she did not verbally encourage the child to select either one or the other puppet, we could not be sure that she did not influence the participant.

*Participant feel asleep (n = 1).* The participant fell asleep during the habituation procedure, and had to be awoken for the test.

**Analyses including excluded infants**

Out of the excluded participants in Study, 6 infants produced an identifiable choice: the participants excluded because of technical failure (1; chose the hinderer), procedural error (3; all three chose the helper), parental interference (1; chose the hinderer) and falling asleep (1; chose the helper). When we included these participants in our data analysis, infants did not significantly prefer the Helper over the Hinderer (16 infants out of 30: *p* = .855, binomial test). When reanalyzing the data for Studies 1 and 2 pooled together, including all participants that produced a choice, we found that 55.5% of infants preferred the Helper over the Hinderer (35/63, *p* = .450, binomial test).
